# Cellular dynamics and molecular signaling networks of plant cytokinesis

**DOI:** 10.1016/j.mocell.2025.100302

**Published:** 2025-12-11

**Authors:** Jiwon Choi, Geert De Jaeger, Hoo Sun Chung

**Affiliations:** 1 Plant Biotechnology Research Center, Ghent University Global Campus, Incheon 21985, Korea; 2 Department of Plant Biotechnology and Bioinformatics, Ghent University, 9052 Ghent, Belgium; 3 VIB Center for Plant Systems Biology, 9052 Ghent, Belgium

**Keywords:** Cell division, Phragmoplast dynamics, Plant cytokinesis, Protein kinase signaling, Spatiotemporal regulation

## Abstract

Cytokinesis, the final stage of cell division, physically partitions the cytoplasm between daughter cells through mechanisms evolved to accommodate unique cellular constraints. Plant cells divide by the formation of rigid cell walls using the phragmoplast—a specialized structure guiding centrifugal cell plate formation from the cell center outward. Despite structural differences from the animal contractile ring mechanism, plant and animal cytokinesis share fundamental similarities in division plane determination, vesicle trafficking, and conserved proteins, including kinesins and microtubule-associated proteins. This conservation alongside kingdom-specific adaptations makes plant cytokinesis an excellent model for understanding evolutionary divergence. Recent technological advances have enabled detailed characterization of molecular components and regulatory networks controlling spatiotemporal progression through post translational modifications. In this review, we provide an integrated perspective of plant cytokinesis, examining cellular dynamics from division plane determination to cell plate maturation, molecular machinery driving these processes, and kinase-mediated regulatory networks ensuring precise coordination of this complex process.

## INTRODUCTION

Cell division is fundamental to all living organisms’ growth, development, and reproduction. The final stage of this process—cytokinesis—physically divides the cytoplasm of the mother cell into two daughter cells following equal distribution of genetic material. In multicellular organisms, cytokinesis takes on expanded significance beyond simple cell multiplication. It orchestrates tissue development through oriented divisions, contributes to cell differentiation via asymmetric divisions, and ensures proper tissue organization. Throughout evolution, organisms have developed diverse mechanisms to accomplish this critical task, suited to their unique cellular structures and constraints.

Animal cells accomplish division through an actomyosin-based contractile ring that constricts inward from the cell periphery. Along the central spindle, filamentous actin and myosin II assemble as a contractile ring under the plasma membrane, while vesicles traffic along the midbody to build the division boundary (Albertson et al., 2008, Guo and Kemphues, 1996, Hu et al., 2012, Nunes et al., 2020, Pardo and Nurse, 2003). In contrast, plant cells face a unique challenge due to their rigid cell walls, which prevent such constriction. Plants have evolved a specialized structure called the phragmoplast to build a new cell plate that grows outward from the center of the cell. The phragmoplast consists of antiparallel microtubules (MTs), actin filaments, and associated proteins. As cytokinesis progresses, the structure develops from cylindrical to ring-like, expanding centrifugally as the new cell plate forms through vesicle deposition (Lipka et al., 2015; Otegui and Staehelin, 2004; Segui-Simarro et al., 2004; Smertenko, 2018; Smertenko et al., 2000; Smertenko et al., 2018).

Despite these structural and mechanical differences, animal and plant cytokinesis share remarkable similarities: both utilize the central spindle to define the division plane after chromosome segregation, rely on vesicle trafficking along antiparallel MTs to build new cell boundaries, and employ highly conserved proteins, including kinesins, microtubule-associated proteins (MAPs), and dynamins. These shared features alongside kingdom-specific adaptations make cytokinesis an excellent model for studying evolutionary divergence in essential cellular processes (Bednarek and Backues, 2010, Ebina et al., 2019, Fujimoto and Tsutsumi, 2014, Heese et al., 2001, Ingouff et al., 2005, Low et al., 2003, Pietra et al., 2013, Shrestha et al., 2012, Swan et al., 1998, Thawani et al., 2018, Thompson et al., 2002, Twell et al., 2002, Whittington et al., 2001).

In this review, we focus on the specialized machinery and molecular regulation of plant cytokinesis. We first outline the cellular dynamics from division plane determination to cell plate formation. We then explore the molecular components that comprise the cytokinesis machinery. Successful cytokinesis requires precise temporal and spatial coordination of these components through post-translational modifications. We review the key protein regulatory networks, with special attention to the protein kinases as critical regulators. Understanding these mechanisms not only reveals plant-specific adaptations but also provides insights into fundamental principles of cellular division across eukaryotes.

## CELLULAR DYNAMICS OF PLANT CYTOKINESIS

**Fig. 1:**
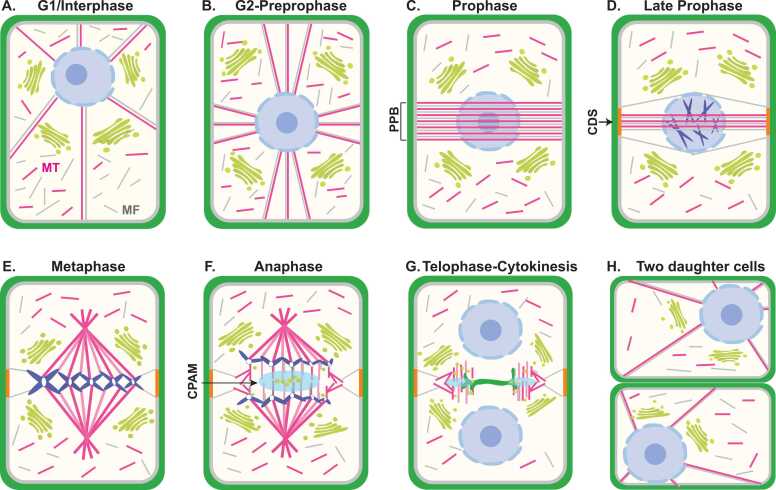
*Cellular dynamics of plant cytokinesis through the cell cycle.* (A) *G1/Interphase:* Cortical microtubules (MT, pink) and microfilaments (MF, gray) are randomly organized around the cell periphery. Golgi stacks (green) are dispersed throughout the cytoplasm. (B) *G2*—*Preprophase:* Nuclear migration to the cell center is guided by the phragmosome, a cytoskeletal network of microtubules and microfilaments radiating from the nucleus toward the cell cortex. (C) *Preprophase:* The preprophase band (PPB) forms as a dense cortical ring of microtubules and microfilaments encircling the nucleus, precisely marking the future division plane where the new cell wall will attach to the parental cell wall. (D) *Late prophase:* PPB disassembles during nuclear envelope breakdown, leaving the cortical division site (CDS, orange bars) marked by molecular landmarks at the cell cortex. (E) *Metaphase:* The mitotic spindle forms with chromosomes aligned at the metaphase plate. The CDS remains marked at the cell cortex where the future cell plate will attach. (F) *Anaphase:* Following chromosome segregation, the spindle midzone begins reorganization and vesicles start accumulating to form the cell plate assembly matrix (CPAM, light-blue region) where the initial cell plate will form. (G) *Telophase*—*Cytokinesis:* The phragmoplast forms from reorganized spindle microtubules and expands centrifugally toward the CDS. Vesicles fuse at the midzone to form the initial cell plate (green), which grows outward to connect with the parental cell wall at the predetermined division sites. (H) *Two daughter cells:* Cytokinesis completes with cell plate fusion to the parental cell wall at the CDS, creating two independent daughter cells with the newly synthesized cell wall.

Plant cells face unique challenges during cytokinesis due to their rigid cell walls, which prevent the contractile ring-based division mechanism used by animal cells. Instead, plants have evolved specialized structures and processes to execute cell division (Fig. 1). This section outlines the chronological progression of plant cytokinesis from division plane determination to cell plate formation.

### Division Plane Determination

In plant cells, the division plane must be precisely determined early in the cell cycle to ensure the correct partitioning of cellular components between daughter cells. This process begins during G2 and early prophase with the migration of the nucleus to the center of the cell, guided by a network of MTs and microfilaments, forming a cytoplasmic structure known as the phragmosome (Mineyuki and Furuya, 1980, Nebenführ et al., 2000). The phragmosome, composed of reorganized cytoskeletal elements and cleared cytoplasm, spans from the nucleus to the cell cortex and helps position the nucleus in the future division plane (Fig. 1A and B).

Following nuclear positioning, a specialized cortical cytoskeletal array called the preprophase band (PPB) forms as a ring of MTs and microfilaments encircling the nucleus (Mineyuki and Palevitz, 1990, Murata and Wada, 1991; Fig. 1C). Initially broad, the PPB progressively narrows during prophase, marking the precise location on the cortex where the new cell wall will eventually fuse with the parental cell wall. This narrowing process involves changes in MT stability and organization by various regulatory proteins and enzymes that control cytoskeletal dynamics (Dahiya and Bürstenbinder, 2023).

Unlike animal cells, which determine their division plane based on spindle orientation during anaphase, plant cells commit to their division site much earlier. The PPB disassembles during nuclear envelope breakdown at the late prophase, but its position is “memorized” at the cell cortex by molecular landmarks collectively known as the cortical division site (CDS) (Müller et al., 2009, Van Damme and Geelen, 2008; Fig. 1D). This positional memory guides the cortical insertion of the new cell plate during cytokinesis.

### Spindle Formation and Chromosome Segregation

After PPB disassembly, the mitotic spindle aligns with the previously defined PPB location, ensuring continuity in spatial orientation. The PPB contributes to spindle orientation by marking the CDS, thus indirectly influencing spindle positioning and axis (Ambrose and Cyr, 2008, Schaefer et al., 2017; Fig. 1E). While plant cells lack centrosomes, the PPB and associated CDS act as positional cues for spindle orientation, playing a role analogous to that of centrosomes in animal cells (Liu and Lee, 2022). The bipolar spindle guides chromosome alignment and segregation during metaphase and anaphase, establishing the spindle midzone that later gives rise to the phragmoplast, a plant-specific structure that mediates cytokinesis (Lee and Liu, 2013).

### Phragmoplast Formation and Expansion

Following chromosome segregation in late anaphase, the phragmoplast forms from the reorganization of spindle MTs. This plant-specific structure consists of 2 sets of antiparallel MTs with their plus ends oriented toward the future division plane. Initially cylindrical, the phragmoplast forms between the daughter nuclei and expands centrifugally toward the CDS in a coordinated manner (Lee and Liu, 2013, Rasmussen et al., 2011; Fig. 1F and G).

As the phragmoplast expands, it undergoes continuous remodeling. MTs disassemble in the central region where the initial cell plate has formed, while new MTs assemble at the leading outer edges. This dynamic turnover produces a ring-like structure that guides the expanding cell plate toward the previously defined division sites at the cell cortex. The balance between MT polymerization at the leading edge and depolymerization at the trailing edge is tightly regulated to maintain phragmoplast structure during expansion and ensure accurate cell plate insertion (Austin et al., 2005; Smertenko, 2018; Smertenko et al., 2011).

### Cell Plate Expansion and Maturation

The phragmoplast serves as a framework for vesicle trafficking from the trans-Golgi network to the division plane. These vesicles carry cell wall materials and membrane components necessary for building the new cell plate. Vesicles fuse at the cell plate initiation site—located at the phragmoplast midzone—to form a tubulo-vesicular network. This intermediate structure matures into a planar fenestrated sheet, then into a tubular network, and eventually into a complete new cell wall (Sinclair et al., 2022; Fig. 1G and H).

The central synthesis of the cell plate requires continuous coordination between vesicle delivery, membrane fusion, and cell wall material deposition. Callose serves as a transient scaffold that stabilizes the early cell plate and is largely degraded later, making way for deposition of cellulose, hemicellulose, and pectins. As the expanding cell plate reaches the parental cell wall at the CDS, it fuses with the plasma membrane, completing the physical separation of the daughter cells (Miart et al., 2014, Sinclair et al., 2022).

**Fig. 2:**
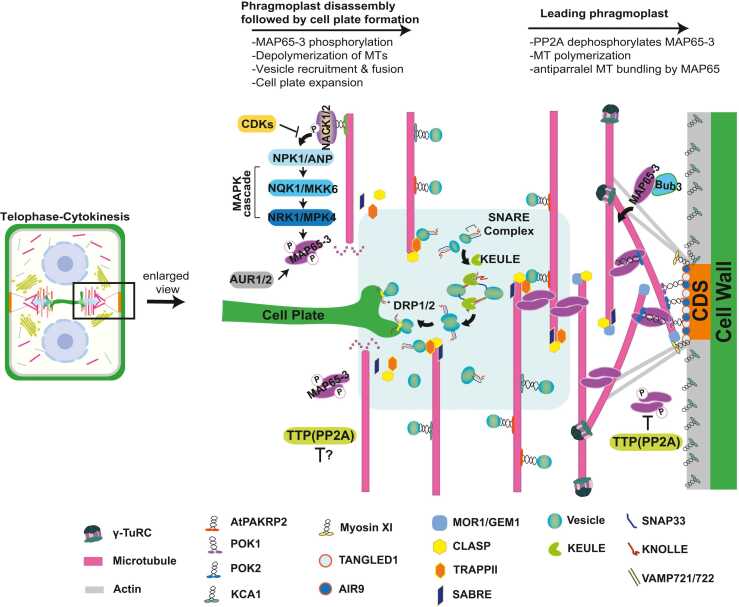
*Molecular regulation of phragmoplast dynamics during plant cytokinesis.* The figure illustrates the coordinated molecular mechanisms controlling phragmoplast expansion and disassembly during cell plate formation. The central light-blue region represents the cell plate assembly matrix (CPAM) where vesicle fusion occurs to build a new cell plate. Right side depicts phragmoplast expansion: γ-TuRC nucleates new microtubules at the leading edge, which are stabilized by MOR1/GEM1 and CLASP. PP2A complex dephosphorylates MAP65. Dephosphorylated MAP65 cross-links antiparallel MTs and guides phragmoplast expansion toward the cortical division site (CDS) through interaction with POK1/2. TANGLED1 and AIR9 provide additional guidance at the CDS where they interact with Myosin XI that persists to maintain division site memory. Bub3 colocalized with MAP65-3 to regulate proper organization of MTs. Left side shows phragmoplast disassembly mechanisms: CDKs initially phosphorylate and inhibit NACK1/2, preventing premature MAPK cascade activation. When CDK activity decreases, this inhibition is relieved, allowing NACK1/2 to activate the sequential MAPK cascade: NPK1/ANP (MAPKKK)-NQK1/MKK6 (MAPKK)-NPK1/MPK4 (MAPK). Activated MPK4 phosphorylates MAP65, reducing their MT-bundling activity and promoting MT turnover at the trailing edge of the expanding phragmoplast. Aurora kinases (AUR1/2) also phosphorylate MAP65-3 proteins to control MT dynamics. It is not clear whether TTP (PP2A) phosphatase regulates the activity of involved protein kinases and how its activity is suppressed in the phragmoplast disassembly zone. Upon MTs destabilization, AtPAKRP2 transports Golgi-derived vesicles along MTs, with TRAPPII facilitating vesicles targeting to the midzone. Membrane fusion is mediated by SNARE complexes containing KNOLLE, SNAP33, and VAMP721,722 with KEULE facilitating complex assembly. DRP1,2 assist in membrane remodeling during cell plate maturation. The process continues until the expanding cell plate reaches and fuses with the parental cell wall at the CDS, completing cytokinesis and creating 2 independent daughter cells.

The precise guidance of the growing cell plate to the predetermined CDS ensures that the new cell wall forms exactly where the PPB was positioned earlier in the cell division, demonstrating the remarkable spatial coordination in plant cytokinesis. As the cell plate nears completion, the central region of the phragmoplast depolymerizes, and the remaining MTs take on a ring-like architecture at the periphery. The ring gradually disassembles as final fusion occurs along the CDS (Sinclair et al., 2022, Smertenko, 2018; Fig. 2).

## MOLECULAR COMPONENTS OF THE CELL DIVISION MACHINERY

The cellular dynamics of plant cytokinesis is driven by the coordinated actions of numerous proteins with distinct functions. This section examines the key molecular components that establish and maintain the division structures through the sequential stages of cytokinesis (Table 1 and Fig. 2).

**Table 1:** Key molecular components and their functions in plant cytokinesis

Cellular events	Key proteins	Molecular function	Cellular function	References
*Division plane determination*				
Nucleus migration	Microtubules (MTs), microfilaments (MFs)	Cytoskeletal framework	Positions the nucleus at cell center	Mineyuki and Furuya (1980); Murata and Wada (1991); Nebenführ et al. (2000)
PPB formation	γ-TURC (γ-tubulin, GCP2, GCP3, and GCP4)	MT nucleation	Initiates cortical MT array formation	Kong et al. (2010); Nakamura et al., 2010, Nakamura et al., 2012; Seltzer et al. (2007)
	MOR1/GEM1	MT stabilization	Organize and stabilize MTs in PPB	Kawamura et al. (2005);Twell et al. (2002); Whittington et al. (2001)
	CLASP	MT stabilization	Coordination of MTs in PPB	Ambrose et al. (2007); Pietra et al. (2013)
	MAP65 family (MAP65-1, -3, -4, -5, -6)	MT stabilization	Organize PPB MTs into parallel arrays	Gaillard et al. (2008); Fache et al. (2010); Smertenko et al., 2004, Smertenko et al., 2006; Caillaud et al. (2008); Li et al. (2017); Van Damme et al. (2004)
PPB maturation	TON2/FASS	TTP complex, Ser/Thr phosphatase	Regulates TON1a and 1b	Camilleri et al. (2002); Kirik et al. (2012); Spinner et al. (2013)
	TON1a, TON1b	TTP complex, MT stabilization	Stabilize and organize PPB MTs	Azimzadeh et al. (2008); Drevensek et al. (2012); Spinner et al. (2013)
	TON1-recruiting motif (TRM)	TTP complex	Target TON1 to PPB MTs	Drevensek et al. (2012); Spinner et al. (2013)
	KATANIN	MT severing	Removes disordered MTs, narrows PPB	McNally and Vale (1993); Komis et al. (2017)
	CLASP	MT binding	MT reorganization in PPB	Ambrose et al. (2007); Pietra et al. (2013)
	SABRE	MT stabilization	MT organization in PPB	Ambrose et al. (2007); Pietra et al. (2013)
CDS maintenance	TANGLED1 (TAN1)	MT binding	Defines CDS (positive marker)	Lipka et al. (2014); Mills et al. (2022); Smith et al. (1996); Walker et al. (2007)
	POK1, POK2	Kinesin motor proteins	Defines CDS (positive marker)	Lipka et al. (2014); Mills et al. (2022); Müller et al. (2006); Herrmann et al. (2018)
	Myosin XI	Actin-based motor proteins	Assembles into CAMPs at PPB site for division site memory	Huang et al. (2024)
	AIR9	MT binding	Defines CDS (positive marker)	Buschmann et al. (2015); Mills et al. (2022)
	KCA1	Kinesin motor protein	Defines CDS (negative marker)	Vanstraelen et al., 2004, Vanstraelen et al., 2006
	Actin MFs	Cytoskeletal element	Defines CDS (negative marker)	Sano et al. (2005); Hoshino et al. (2003)
*Phragmoplast assembly and dynamics*				
Initial assembly and MT organization	γ-TuRC	MT nucleation	Initiates new MTs polymerization for phragmoplast	Kong et al. (2010); Murata et al. (2013)
	MAP65-3	MT bundling	Cross-links MTs and interacts with POK	Boruc et al. (2016); Herrmann et al. (2018); Ho et al. (2011); Kosetsu et al. (2013); Sasabe et al. (2011b)
	MOR1/GEM1	MT stabilization	Phragmoplast expansion	Kawamura et al. (2005);Twell et al. (2002); Whittington et al. (2001)
	AtPAKRP1, AtPAKRP1L	Plus-end kinesin	Phragmoplast expansion	Lee et al., 2001, Lee et al., 2007; Pan et al. (2004)
	KCBP, KCA1, and KCA2	Minus-end kinesin	Phragmoplast expansion	Buschmann et al. (2015); Vanstraelen et al., 2004, Vanstraelen et al., 2006
	AIR9	MT binding	Guides phragmoplast to CDS	Buschmann et al. (2015)
	CLASP	MT plus-end binding	Prevents MT depolymerization at plus ends	Ambrose et al. (2007)
	POK1/2	Kinesin, MT stabilization	Guides phragmoplast to CDS	Müller et al. (2006)
	TANGLED1 (TAN1)	MT binding	Guides phragmoplast to CDS	Walker et al. (2007)
Phragmoplast dynamics	CDKs	Phosphorylate NACK1 and inhibit its interaction with NPK1, enhance *KNOLLE* transcription	Regulates phragmoplast MTs stability	Haga et al. (2007); Sasabe et al. (2011a)
	APC complex	E3 ligase	Degrades cyclin B1	Guo et al. (2016)
	AtNACK1/HINKEL, AtNACK2/TETRASPORE	Kinesin motor protein, activates MPK4 cascade	Regulates phragmoplast expansion	Nishihama et al. (2001);Tanaka et al. (2004); Takahashi et al. (2010)
	ANP1,2,3 (NPK1 in tobacco)	MAPKKK, activates MPK4 cascade	Regulates phragmoplast MT turnover	Krysan et al. (2002);Takahashi et al. (2010); Nishihama et al. (2001)
	MKK6 (NQK1 in tobacco)	MAPKK, activates MPK4 cascade	Regulates phragmoplast MT turnover	Soyano et al. (2003);Takahashi et al. (2010)
	MPK4 (NRK1 in tobacco)	Phosphorylates MAP65	Regulates phragmoplast MT turnover	Beck et al. (2011); Kosetsu et al. (2010); Sasabe et al. (2006); Takahashi et al. (2010)
	AUR1, AUR2	Regulation of MTs stability	Regulates MAP65-3 at phragmoplast	Boruc et al. (2016); Deng et al. (2024); Kawabe et al. (2005); Petrovská et al. (2012); Van Damme et al. (2011)
	TPX2, TPXL	AUR1 regulators	Target AUR1 to phragmoplast MTs	Boruc et al. (2019); Petrovská et al. (2013)
	MAP65-3	MT bundling	Reorganize phragmoplast structure	Ho et al. (2011); Steiner et al. (2016); Herrmann et al. (2018)
	BUB3	Spindle assembly checkpoint protein	Phragmoplast MT organization	Zhang et al. (2018)
*Cell plate formation*				
Vesicle targeting and tethering	TRAPPII complex (TRS120,130/CLUB)	Vesicle recruitment	Vesicle transport to midzone	Qi et al. (2011)
	MAP65-3	Scaffolding	Links vesicles to MTs via TRAPII	Steiner et al. (2016)
	AtPAKRP2	Kinesin motor protein	Transports vesicles along MTs	Lee et al. (2001)
	Exocyst complex (SEC3/5/6/8/10/15, EXO70/84)	Vesicle transport	Tethers and positions vesicles for fusion	Chong et al. (2010); Wu et al. (2013)
Membrane fusion and remodeling	KNOLLE	t-SNARE (syntaxin)	Mediates vesicle fusion at cell plate	Batoko and Moore (2001); Lauber et al. (1997)
	SNAP33	t-SNARE protein	Forms fusion complex with KNOLLE	El Kasmi et al. (2013)
	VAMP721,722	v-SNARE proteins	Mediates vesicle fusion	Zhang et al. (2011)
	KEULE	SM protein	Facilitates SNARE complex assembly	Wu et al. (2013); Steiner et al. (2016)
	MYB3R4	Transcription factor	Activates *KNOLLE* transcription	Haga et al. (2007)
	DRP1, DRP2	Dynamins, GTPase	Remodels fused vesicle membranes	Bednarek and Backues (2010)

### Division Plane Establishment Components

The establishment and maintenance of the division plane involve several key protein complexes. During PPB formation, MT organization depends on nucleation factors such as γ-tubulin ring complex, composed of γ-tubulin and γ-tubulin complex proteins (GCPs), which initiates new MT growth at the cell cortex (Kong et al., 2010, Nakamura et al., 2010, Nakamura et al., 2012, Seltzer et al., 2007). MAPs, including Microtubule Organization 1 (MOR1)/Gemini Pollen 1, Cytoplasmic Linker Protein-Associated Protein (CLASP), SABRE, and Microtubule-Associated Protein 65 (MAP65) family members, stabilize and organize these cortical MTs into the band structure (Ambrose et al., 2007, Ho et al., 2011, Kawamura et al., 2005, Kirik et al., 2007, Li et al., 2017, Pietra et al., 2013, Twell et al., 2002, Van Damme et al., 2004, Whittington et al., 2001). CLASP interacts with SABRE to stabilize MTs bundled by MAP65 proteins. MOR/Gemini Pollen 1 binds to MTs and establishes precisely ordered arrays of cortical MTs. Together, these proteins facilitate the proper alignment of MTs within the PPB.

The narrowing and maturation of the PPB involves a cortical scaffold comprising TONNEAU1 (TON1) and TON1-recruiting motif (TRM) proteins, which together recruit the regulatory subunit TON2/FASS of protein phosphatase 2A (PP2A) to the cell cortex. This TON1-TRM-PP2A module coordinates spatial and temporal MT organization necessary for proper PPB formation and division plane determination (Azimzadeh et al., 2008, Camilleri et al., 2002, Drevensek et al., 2012, Kirik et al., 2012, Spinner et al., 2013). Although direct substrates are not identified yet, this indicates importance of protein kinase dynamics for proper MT organization. Additionally, the MT-severing protein KATANIN together with MT-stabilizing proteins CLASP and SABRE promotes PPB formation by severing and reorganizing MTs and, thereby shaping the band into its narrow, precise form (Kirik et al., 2007, Komis et al., 2017, McNally and Vale, 1993, Pietra et al., 2013).

After PPB disassembly, the division plane is marked by both positive and negative molecular cues. Positive markers include TANGLED1 (TAN1), PHRAGMOPLAST ORIENTING KINESIN 1 and 2 (POK1, POK2) together with AUXIN-INDUCED IN ROOT CULTURES9 (AIR9), which remain at the CDS throughout mitosis, guiding the expanding phragmoplast toward its correct position (Lipka et al., 2014, Müller et al., 2006, Mills et al., 2022, Smith et al., 1996, Smith et al., 2001, Walker et al., 2007; Fig. 2). TAN1 recruits POK1/2 and AIR9 to the division site to directly mediate MT interaction to the site. These positive markers work in coordination with dynamic cytoskeletal rearrangements that refine division site memory. During PPB formation, MTs recruit Myosin XI and Kinesin-12 motor proteins to the CDS, where they assemble into Cytoskeleton-Associated Motor assemblies at the PPB site (CAMPs), a persistent scaffold that remains throughout mitosis to provide continuous spatial information guiding phragmoplast expansion toward the predetermined attachment site (Huang et al., 2024). Complementing these positive cues, negative markers create exclusion zones, such as the KINESIN CDKA-ASSOCIATED1 (KCA1)-depleted zone (KDZ), where KCA1 is absent (Vanstraelen et al., 2004, Vanstraelen et al., 2006), and the actin-depleted zone (ADZ), formed by local depolymerization of cortical actin filaments (Hoshino et al., 2003, Sano et al., 2005). These exclusion zones help accurately guide the expanding phragmoplast by defining the regions where the cell plate will attach to the parental cell wall. In the absence of these markers, misoriented cell plates are frequently observed, highlighting their essential role in spatial guidance.

### Phragmoplast Assembly and Dynamics

The phragmoplast MT array is organized by several key proteins. MAP65 family members, particularly MAP65-3/PLEIADE, cross-link antiparallel MTs and interact with POKs to guide the phragmoplast toward the CDS (Herrmann et al., 2018, Ho et al., 2011, Kosetsu et al., 2013, Sasabe et al., 2011b). The continuous assembly of new MTs at the expanding phragmoplast edge depends on γ-tubulin ring complex nucleation complexes and the MT-stabilizing protein Microtubule Organization 1, which promotes polymerization of new MTs at the leading edge (Kawamura et al., 2005, Kong et al., 2010, Murata et al., 2005, Twell et al., 2002).

Multiple kinesin motor proteins contribute to organization and function of the phragmoplast. The kinesin-12 family members, Kinesin-12A/PAKRP1 and Kinesin-12B/PAKRP1L, establish the interdigitating MT arrays near the forming cell plate through interactions with plus-end MTs (Lee and Liu, 2013; Lee et al., 2001, 2007; Pan et al., 2004). The kinesin-14 family includes calmodulin-binding kinesin-like protein, which regulates minus-end MTs through AIR9 interaction, and KCA1/KCA2, which also associate with minus-end MTs (Buschmann et al., 2015, Vanstraelen et al., 2004, Vanstraelen et al., 2006). Kinesin-7 members, NPK1-activating kinesin-like protein AtNACK1/HINKEL, and AtNACK2/TETRASPORE deliver regulatory signals that control MT turnover during phragmoplast expansion, affecting the interaction between MTs and MAP65 proteins (Nishihama and Machida, 2001; Soyano et al., 2003; Tanaka et al., 2004). Furthermore, MT-binding protein TAN1 guides phragmoplast expansion to the CDS through interaction with POK1 and POK2 (Müller et al., 2006, Walker et al., 2007).

### Vesicle Trafficking and Cell Plate Formation

Construction of the new cell plate depends on precise vesicle trafficking and membrane fusion. Golgi-derived vesicles carrying cell wall precursors travel along phragmoplast MTs to the division plane. MAP65-3/PLEIADE facilitates vesicle localization at the midzone via interactions with Kinesin-12 and the vesicle-tethering factor, Transport Protein Particle II complex (Ho et al., 2011; Lee et al., 2001, 2007; Qi et al., 2011; Steiner et al., 2016). The octameric exocytosis complex, including subunits of SEC3, SEC5, SEC6, SEC8, SEC10, SEC15, EXO70, and EXO84, tethers these vesicles to their destination, preparing them for fusion with the developing cell plate (Chong et al., 2010, Sinclair et al., 2022, Wu et al., 2013).

Membrane fusion is mediated by soluble N-ethylmaleimide-sensitive factor attachment receptor (SNARE) proteins, with the cytokinesis-specific syntaxin KNOLLE playing an essential role. KNOLLE interacts with other SNARE proteins, including soluble N-ethylmaleimide-sensitive factor adaptor protein 33 and Vesicle-Associated Membrane Protein721 and VAMP722 to form a fusion complex, as well as with KEULE, which facilitates SNARE complex assembly (Batoko and Moore, 2001; El Kasmi et al., 2013; Lauber et al., 1997; Zhang et al., 2011). Additionally, dynamin-related proteins, DRP1 and DRP2, assist in membrane remodeling during cell plate maturation (Bednarek and Backues, 2010).

Recent studies have revealed critical roles for anionic lipids, particularly phosphatidylinositol-4-phosphate and phosphatidylinositol-4,5-bisphosphate (PI(4,5)P₂), in regulating cell plate membrane organization and morphology during cytokinesis (Lebecq et al., 2023, Luo et al., 2025). The phosphatase SAC9 establishes spatial polarity by depleting PI(4,5)P₂ at the cell plate leading zone, which guides proper cell plate attachment and prevents ectopic recruitment of cytokinesis machinery, while phosphatidylinositol-4-phosphate inhibits the flippase ALA1 to control phosphatidylserine levels and membrane curvature, and PI(4,5)P₂ recruits the dynamin-related protein DRP1A to promote constriction region formation. Together, these phosphoinositide-mediated mechanisms coordinate transbilayer lipid asymmetry, membrane remodeling, and the spatial separation of cell plate expansion from plasma membrane identity acquisition, ensuring successful completion of plant cell division.

The nascent cell plate undergoes maturation through the actions of various cell wall-synthesizing enzymes. Initially forming as a tubular-vesicular network, the structure matures into a tubular network through membrane consolidation and callose deposition, eventually developing into a complete cell wall as additional components are added (Miart et al., 2014, Segui-Simarro et al., 2004, Sinclair et al., 2022). This orchestrated process ensures the formation of a structurally sound cell wall that completes the physical separation of the daughter cells.

## POST TRANSLATIONAL REGULATION OF CYTOKINESIS

The dynamic rearrangements of MTs during plant cytokinesis require precise spatial and temporal control through post translational modifications, notably kinase-mediated phosphorylation. Three major kinase families—Cyclin-Dependent Kinases (CDKs), mitogen-activated protein kinases (MAPKs), and Auroral Kinases (AURs)—work in concert to regulate distinct yet overlapping aspects of plant cytokinesis.

### Cyclin-Dependent Kinases

CDKs, in complex with cyclins, serve as master regulators of cell cycle progression. During plant cytokinesis, CDK activity must be precisely regulated—high activity is required for mitotic entry, while reduction is necessary for successful cytokinesis. The plant-specific kinase CDKB1, in complex with cyclin B, promotes the G2/M transition (Porceddu et al., 2001). The timely degradation of cyclin B by the Anaphase-Promoting Complex/Cyclosome (APC/C) is critical for reducing CDK activity during the transition from metaphase to cytokinesis (Guo et al., 2016). The APC/C, an E3 ubiquitin ligase, is sequentially activated by CDC20 during metaphase-anaphase transition and later by CCS52/FZR during cytokinesis. Studies with nondegradable cyclin B mutants exhibit severe phragmoplast disorganization and mislocalization of key cytokinetic proteins such as KNOLLE and NACK1, demonstrating that APC/C-mediated cyclin degradation is essential for proper cytokinesis (Heyman and DeVeylder, 2012; Weingartner et al., 2004).

As CDK activity decreases following cyclin degradation, this relieves the inhibitory phosphorylation of the MAPK pathway components NACK1 and NPK1, leading to activation of the MAPK cascade that promotes phragmoplast expansion (Sasabe et al., 2011a). Additionally, the tightly regulated CDK-cyclin complex controls the transcription factor MYB3R4, which enhances the expression of cytokinesis-specific genes including the syntaxin *KNOLLE* (Haga et al., 2007).

### Mitogen-Activated Protein Kinases Cascade

The MAPK cascade is a conserved 3-tiered signaling system consisting of MAP kinase kinase kinase, MAP kinase kinase (MAPKK), and MAP kinase (MAPK), which regulates myriad cellular processes across eukaryotes (Tena et al., 2001, Xu and Zhang, 2015, Zhang and Zhang, 2022). In plant cytokinesis, this pathway plays a crucial role in controlling phragmoplast expansion and cell plate formation. Activation of the cascade is triggered by kinesin-like proteins such as NACK1/HINKEL, which initiate phosphorylation of MAP kinase kinase kinase (NPK1 in tobacco; ANP1,2,3 in Arabidopsis), sequentially activating MAPKK (NQK1/MKK6) and MAPK (NRK1/MPK4) (Krysan et al., 2002, Sasabe et al., 2006, Takahashi et al., 2010).

In Arabidopsis, this pathway converges on MPK4, which phosphorylates MAP65 family proteins at the phragmoplast. This phosphorylation reduces the MT-bundling activity of MAP65, promoting the necessary MT turnover that allows the phragmoplast expansion toward the cell cortex. Mutants defective in cascade components exhibit severe cytokinetic defects, including incomplete and disorganized cell plates (Beck et al., 2011, Kosetsu et al., 2010, Takahashi et al., 2010). Additionally, phosphatidylinositol-4-kinases β1 and β2 function together with MPK4 to regulate vesicle tracking during cell plate formation (Lin et al., 2019). Collectively, MPK4 plays an important role in spatially and temporally regulating cytoskeletal and vesicle trafficking elements essential for cytokinesis.

### Aurora Kinases

Aurora kinases are evolutionarily conserved regulators of mitosis and cytokinesis. Arabidopsis encodes 3 Aurora kinases: 2 Aurora A-type (AUR1 and AUR2) and one AUR B-type (AUR3) (Kawabe et al., 2005). Among these, AUR1 plays the most prominent role in cytokinesis, by phosphorylating MAP65 proteins to regulate MT dynamics (Boruc et al., 2016, Van Damme et al., 2011). The localization and activity of AUR1 are regulated through interaction with targeting protein for Xklp2 (TPX2) and related proteins (Boruc et al., 2019, Petrovská et al., 2012, Petrovská et al., 2013). The AUR1-TPX2 complex associates with phragmoplast MTs and persists until cytokinesis completion. Notably, even after TPX2 degradation, AUR1 colocalizes with KNOLLE at the cell plate formation site, suggesting AUR1 is involved in both MT organization and membrane trafficking during cytokinesis (Deng et al., 2024). Unlike its animal counterparts, plant AUR1 has evolved specialized functions in phragmoplast organization and cell plate formation, highlighting the adaptation of conserved kinases to plant-specific cytokinetic mechanisms.

## UNANSWERED QUESTIONS AND FUTURE PERSPECTIVES

Despite significant progress in understanding the molecular networks governing plant cytokinesis, fundamental questions remain across multiple aspects of the process. What molecular mechanisms control cytokinetic dynamics, such as phragmoplast expansion and disassembly rates coordinated with cell plate expansion? What additional molecular machinery remains to be discovered, and what determines the spatiotemporal specificity of protein-protein interactions within the phragmoplast? How are multiple kinase signaling pathways interconnected, do they converge on common or specific targets, and how are their actions coordinated in space and time?

The complexity of kinase signaling networks is exemplified by MAPK and Aurora kinase pathways, which both target MAP65 proteins to regulate phragmoplast dynamics, yet their loss-of-function mutants display contrasting cytokinetic phenotypes (Deng et al., 2024). This paradox suggests distinct regulatory roles despite shared substrates, highlighting gaps in our understanding of pathway specificity and crosstalk. Studying kinase function within these pathways has been challenging due to their essential roles, which limit the effectiveness of traditional genetic screens. However, inducible loss-of-function systems combined with specific chemical inhibitors now offer powerful tools to bypass lethality and dissect gene function with temporal precision.

Emerging technologies are increasing our capacity to address these questions. Recent breakthroughs in advanced microscopy techniques, particularly lattice light-sheet microscopy, are revolutionizing the capture of complete cytokinetic events in 4 dimensions (3D space + time) with minimal photobleaching. Advances in imaging, combined with biochemical tools such as phosphoproteomics and protein-protein interaction mapping, are enabling spatiotemporal resolution of cytokinesis regulation. The establishment of quantitative frameworks for tracking phase transitions provides objective benchmarks for dissecting the contributions of kinases, cytoskeletal elements, membrane trafficking machinery, and polysaccharide synthesis enzymes. Moving forward, the integration of lattice light-sheet microscopy and super-resolution imaging with biochemical approaches and inducible genetic systems will be essential to fully elucidate the complex regulatory networks underlying plant cytokinesis. Such integrative approaches promise not only to resolve fundamental mechanistic questions but also to reveal how cytokinetic machinery has been adapted across plant lineages to accommodate diverse developmental strategies and environmental challenges.

## Funding and Support

This study was supported by the Korean National Research Foundation (2021R1F1A1048959), UGent-BOF starting grant (STA202002007) to H.S.C., and Ghent University Global Campus-Research Center 1-Plant Biotechnology Research Center (GUGC-RC1-PBRC) internal research fund.

## Author Contributions

Jiwon Choi and Hoo Sun Chung conceived and designed the review and wrote the manuscript. Geert De Jaeger contributed additional scientific insights and provided critical editorial suggestions.

## Declaration of Competing Interests

The authors declare that they have no known competing financial interests or personal relationships that could have appeared to influence the work reported in this paper.

## References

[bib1] Albertson R., Cao J., Hsieh T.-S., Sullivan W. (2008). Vesicles and actin are targeted to the cleavage furrow via furrow microtubules and the central spindle. J. Cell Biol..

[bib2] Ambrose J.C., Cyr R. (2008). Mitotic spindle organization by the preprophase band. Mol. Plant.

[bib3] Ambrose J.C., Shoji T., Kotzer A.M., Pighin J.A., Wasteneys G.O. (2007). The arabidopsis CLASP gene encodes a microtubule-associated protein involved in cell expansion and division. Plant Cell.

[bib4] Austin J.R., Segui-Simarro J.M., Staehelin L.A. (2005). Quantitative analysis of changes in spatial distribution and plus-end geometry of microtubules involved in plant-cell cytokinesis. J. Cell Sci..

[bib5] Azimzadeh J., Nacry P., Christodoulidou A., Drevensek S., Camilleri C., Amiour N., Parcy F., Pastuglia M., Bouchez D. (2008). Arabidopsis TONNEAU1 proteins are essential for preprophase band formation and interact with centrin. Plant Cell.

[bib6] Batoko H., Moore I. (2001). Plant cytokinesis: KNOLLE joins the club. Curr. Biol..

[bib7] Beck M., Komis G., Ziemann A., Menzel D., Šamaj J. (2011). Mitogen-activated protein kinase 4 is involved in the regulation of mitotic and cytokinetic microtubule transitions in *Arabidopsis thaliana*. New Phytologist.

[bib8] Bednarek S.Y., Backues S.K. (2010). Plant dynamin-related protein families DRP1 and DRP2 in plant development. Biochem. Soc. Trans..

[bib9] Boruc J., Deng X., Mylle E., Besbrugge N., Van Durme M., Demidov D., Tomaštíková E.D., Tan T.C., Vandorpe M., Eeckhout D. (2019). TPX2-LIKE PROTEIN3 is the primary activator of α-aurora kinases and is essential for embryogenesis. Plant Physiol..

[bib10] Boruc J., Weimer A.K., Stoppin-Mellet V., Mylle E., Kosetsu K., Cedeño C., Jaquinod M., Njo M., De Milde L., Tompa P. (2016). Phosphorylation of MAP65-1 by arabidopsis aurora kinases is required for efficient cell cycle progression. Plant Physiol..

[bib11] Buschmann H., Dols J., Kopischke S., Peña E.J., Andrade-Navarro M.A., Heinlein M., Szymanski D.B., Zachgo S., Doonan J.H., Lloyd C.W. (2015). Arabidopsis KCBP interacts with AIR9 but stays in the cortical division zone throughout mitosis via its MyTH4-FERM domain. J. Cell Sci..

[bib12] Caillaud M.C., Lecomte P., Jammes F., Quentin M. l, Pagnotta S., Andrio E., de Almeida Engler J., Marfaing N., Gounon P., Abad P. (2008). MAP65-3 microtubule-associated protein is essential for nematode-induced giant cell ontogenesis in arabidopsis. Plant Cell.

[bib13] Camilleri C., Azimzadeh J., Pastuglia M., Bellini C., Grandjean O., Bouchez D. (2002). The Arabidopsis TONNEAU2 gene encodes a putative novel protein phosphatase 2A regulatory subunit essential for the control of the cortical cytoskeleton. Plant Cell.

[bib14] Chong Y.T., Gidda S.K., Sanford C., Parkinson J., Mullen R.T., Goring D.R. (2010). Characterization of the *Arabidopsis thaliana* exocyst complex gene families by phylogenetic, expression profiling, and subcellular localization studies. New Phytol..

[bib15] Dahiya P., Bürstenbinder K. (2023). The making of a ring: assembly and regulation of microtubule-associated proteins during preprophase band formation and division plane set-up. Curr. Opin. Plant Biol..

[bib16] Deng X., Xiao Y., Tang X., Liu B., Lin H. (2024). Arabidopsis α Aurora kinase plays a role in cytokinesis through regulating MAP65-3 association with microtubules at phragmoplast midzone. Nat. Commun..

[bib17] Drevensek S., Gousso M., Duroc Y., Christodoulidou A., Steyaert S., Schaefer E., Duvernois E., Grandjean O., Vantard M., Bouchez D. (2012). The Arabidopsis TRM1-TON1 interaction reveals a recruitment network common to plant cortical microtubule arrays and eukaryotic centrosomes. Plant Cell.

[bib18] Ebina H., Ji L., Sato M. (2019). CLASP promotes microtubule bundling in metaphase spindle independently of Ase1/PRC1 in fission yeast. Biol. Open.

[bib19] El Kasmi F., Krause C., Hiller U., Stierhof Y.D., Mayer U., Conner L., Kong L., Reichardt I., Sanderfoot A.A., Jürgens G. (2013). SNARE complexes of different composition jointly mediate membrane fusion in Arabidopsis cytokinesis. Mole. Biol. Cell.

[bib20] Fache V., Gaillard J., Van Damme D., Geelen D., Neumann E., Stoppin-Mellet V., Vantard M. (2010). Arabidopsis kinetochore fiber-associated MAP65-4 cross-links microtubules and promotes microtubule bundle elongation. Plant Cell.

[bib21] Fujimoto M., Tsutsumi N. (2014). Dynamin-related proteins in plant post-Golgi traffic. Front. Plant Sci..

[bib22] Gaillard J., Neumann E., Van Damme D., Stoppin-Mellet V., Ebel C., Barbier E., Geelen D., Vantard M. (2008). Two microtubule-associated proteins of Arabidopsis MAP65s promote antiparallel microtubule bundling. Mol. Biol. Cell.

[bib23] Guo L., Jiang L., Zhang Y., Lu X.L., Xie Q., Weijers D., Liu C.M. (2016). The anaphase-promoting complex initiates zygote division in Arabidopsis through degradation of cyclin B1. Plant J.

[bib24] Guo S., Kemphues K.J. (1996). A non-muscle myosin required for embryonic polarity in *Caenorhabditis elegans*. Nature.

[bib25] Haga N., Kato K., Murase M., Araki S., Kubo M., Demura T., Suzuki K., Müller I., Voß U., Jürgens G. (2007). R1R2R3-Myb proteins positively regulate cytokinesis through activation of KNOLLE transcription in *Arabidopsis thaliana*. Development.

[bib26] Heese M., Gansel X., Sticher L., Wick P., Grebe M., Granier F., Jürgens G. (2001). Functional characterization of the KNOLLE-interacting t-SNARE AtSNAP33 and its role in plant cytokinesis. J Cell Biol.

[bib27] Herrmann A., Livanos P., Lipka E., Gadeyne A., Hauser M.T., Van Damme D., Müller S. (2018). Dual localized kinesin-12 POK2 plays multiple roles during cell division and interacts with MAP65-3. EMBO Rep.

[bib28] Heyman J., De Veylder L. (2012). The anaphase-promoting complex/cyclosome in control of plant development. Mol. Plant.

[bib29] Ho C.M.K., Hotta T., Guo F., Roberson R.W., Lee Y.R.J., Liu B. (2011). Interaction of antiparallel microtubules in the phragmoplast is mediated by the microtubule-associated protein MAP65-3 in Arabidopsis. Plant Cell.

[bib30] Hoshino H., Yoneda A., Kumagai F., Hasezawa S. (2003). Roles of actin-depleted zone and preprophase band in determining the division site of higher-plant cells, a tobacco BY-2 cell line expressing GFP-tubulin. Protoplasma.

[bib31] Hu C.-K., Coughlin M., Mitchison T.J. (2012). Midbody assembly and its regulation during cytokinesis. Mole. Biol. Cell.

[bib32] Huang C.H., Peng F.L., Lee Y.J., Liu B. (2024). The microtubullarpreprophase band recruits Myosin XI to the cortical division site to guide phragmoplast expansion during plant cytokinesis. Dev. Cell.

[bib33] Ingouff M., Gerald J.N.F., Guérin C., Robert H., Sørensen M.B., Damme D.V., Geelen D., Blanchoin L., Berger F. (2005). Plant formin AtFH5 is an evolutionarily conserved actin nucleator involved in cytokinesis. Nat. Cell Biol..

[bib34] Kawabe A., Matsunaga S., Nakagawa K., Kurihara D., Yoneda A., Hasezawa S., Uchiyama S., Fukui K. (2005). Characterization of plant Aurora kinases during mitosis. Plant Mole. Biol..

[bib35] Kawamura E., Himmelspach R., Rashbrooke M.C., Whittington A.T., Gale K.R., Collings D.A., Wasteneys G.O. (2005). Microtubule organization 1 regulates structure and function of microtubule arrays during mitosis and cytokinesis in the Arabidopsis root. Plant Physiol.

[bib36] Kirik A., Ehrhardt D.W., Kirik V. (2012). TONNEAU2/FASS regulates the geometry of microtubule nucleation and cortical array organization in interphase Arabidopsis cells. Plant Cell.

[bib37] Kirik V., Herrmann U., Parupalli C., Sedbrook J.C., Ehrhardt D.W., Hülskamp M. (2007). CLASP localizes in two discrete patterns on cortical microtubules and is required for cell morphogenesis and cell division in Arabidopsis. J Cell Sci.

[bib38] Komis G., Luptovčiak I., Ovečka M., Samakovli D., Šamajová O., Šamaj J. (2017). Katanin effects on dynamics of cortical microtubules and mitotic arrays in *Arabidopsis thaliana* revealed by advanced live-cell imaging. Front. Plant Sci..

[bib39] Kong Z., Hotta T., Lee Y.R.J., Horio T., Liu B. (2010). The $\gamma$-Tubulin complex protein GCP4 is required for organizing functional microtubule arrays in *Arabidopsis thaliana*. Plant Cell.

[bib40] Kosetsu K., de Keijzer J., Janson M.E., Goshima G. (2013). Microtubule-associated Protein65 is essential for maintenance of phragmoplast bipolarity and formation of the cell plate in *Physcomitrella patens*. Plant Cell.

[bib41] Kosetsu K., Matsunaga S., Nakagami H., Colcombet J., Sasabe M., Soyano T., Takahashi Y., Hirt H., Machida Y. (2010). The MAP kinase MPK4 is required for cytokinesis in *Arabidopsis thaliana*. Plant Cell.

[bib42] Krysan P.J., Jester P.J., Gottwald J.R., Sussman M.R. (2002). An Arabidopsis mitogen-activated protein kinase kinase kinase gene family encodes essential positive regulators of cytokinesis. Plant Cell.

[bib43] Lauber M.H., Waizenegger I., Steinmann T., Schwarz H., Mayer U., Hwang I., Lukowitz W., Jürgens G. (1997). The Arabidopsis KNOLLE protein is a cytokinesis-specific syntaxin. J. Cell Biol..

[bib44] Lebecq A., Goldy C., Fangain A., Gascon E., Belcram K., Pastuglia M., Bouchez D., Caillaud M.-C. (2023). The phosphoinositide signature guides the final step of plant cytokinesis. Sci. Adv..

[bib45] Lee Y.R.J., Giang H.M., Liu B. (2001). A novel plant Kinesin-related protein specifically associates with the phragmoplast organelles. Plant Cell.

[bib46] Lee Y.R.J., Li Y., Liu B. (2007). Two Arabidopsis phragmoplast-associated kinesins play a critical role in cytokinesis during male gametogenesis. Plant Cell.

[bib47] Lee Y.R., Liu B. (2013). The rise and fall of the phragmoplast microtubule array. Curr. Opin. Plant Biol..

[bib48] Li H., Sun B., Sasabe M., Deng X., Machida Y., Lin H., Lee Y.R.J., Liu B. (2017). Arabidopsis MAP65-4 plays a role in phragmoplast microtubule organization and marks the cortical cell division site. New Phytol..

[bib49] Lin F., Krishnamoorthy P., Schubert V., Hause G., Heilmann M., Heilmann I. (2019). A dual role for cell plate-associated PI4K$\beta$ in endocytosis and phragmoplast dynamics during plant somatic cytokinesis. EMBO J..

[bib50] Lipka E., Gadeyne A., Stöckle D., Zimmermann S., De Jaeger G., Ehrhardt D.W., Kirik V., Van Damme D., Müller S. (2014). The phragmoplast-orienting Kinesin-12 class proteins translate the positional information of the preprophase band to establish the cortical division zone in *Arabidopsis thaliana*. Plant Cell.

[bib51] Lipka E., Herrmann A., Mueller S. (2015). Mechanisms of plant cell division. WIREs Dev. Biol..

[bib52] Liu B., Lee Y.J. (2022). Spindle assembly and mitosis in plants. Annu. Rev. Plant Biol.

[bib53] Low S.H., Li X., Miura M., Kudo N., Quiñones B., Weimbs T. (2003). Syntaxin 2 and endobrevin are required for the terminal step of cytokinesis in mammalian cells. Dev. Cell.

[bib54] Luo Y., Tian Y.-F., Liu H.-R., Yang W.-C. (2025). Phosphatidylinositides regulate the cell plate morphology transition during cytokinesis in *Arabidopsis*. Nat. Commun..

[bib55] McNally F.J., Vale R.D. (1993). Identification of katanin, an ATPase that severs and disassembles stable microtubules. Cell.

[bib56] Miart F., Desprez T., Biot E., Morin H., Belcram K., Höfte H., Gonneau M., Vernhettes S. (2014). Spatio-temporal analysis of cellulose synthesis during cell plate formation in *Arabidopsis*. Plant J..

[bib57] Mills A.M., Morris V.H., Rasmussen C.G. (2022). The localization of PHRAGMOPLAST ORIENTING KINESIN1 at the division site depends on the microtubule-binding proteins TANGLED1 and AUXIN-INDUCED IN ROOT CULTURES9 in Arabidopsis. Plant Cell.

[bib58] Mineyuki Y., Furuya M. (1980). Effect of centrifugation on the development and timing of premitotic positioning of the nucleus in *Adidantum* protonemata. Dev Growth Diff.

[bib59] Mineyuki Y., Palevitz B.A. (1990). Relationship between preprophase band organization, F-actin and the division site in *Allium*: fluorescence and morphometric studies on cytochalasin-treated cells. J. Cell Sci..

[bib60] Müller S., Han S., Smith L.G. (2006). Two kinesins are involved in the spatial control of cytokinesis in *Arabidopsis thaliana*. Curr. Biol..

[bib61] Müller S., Wright A.J., Smith L.G. (2009). Division plane control in plants: new players in the band. Trends Cell Biol.

[bib62] Murata T., Sano T., Sasabe M., Nonaka S., Higashiyama T., Hasezawa S., Machida Y., Hasebe M. (2013). Mechanism of microtubule array expansion in the cytokinetic phragmoplast. Nat. Commun..

[bib63] Murata T., Sonobe S., Baskin T.I., Hyodo S., Hasezawa S., Nagata T., Horio T., Hasebe M. (2005). Microtubule-dependent microtubule nucleation based on recruitment of $\gamma$-tubulin in higher plants. Nat. Cell Biol..

[bib64] Murata T., Wada M. (1991). Effects of centrifugation on preprophase-band formation in *Adiantum* protonemata. Planta.

[bib65] Nakamura M., Ehrhardt D.W., Hashimoto T. (2010). Microtubule and katanin-dependent dynamics of microtubule nucleation complexes in the acentrosomal *Arabidopsis* cortical array. Nat. Cell Biol..

[bib66] Nakamura M., Yagi N., Kato T., Fujita S., Kawashima N., Ehrhardt D.W., Hashimoto T. (2012). *Arabidopsis* GCP3-interacting protein 1/MOZART 1 is an integral component of the $\gamma$-tubulin-containing microtubule nucleating complex. Plant J..

[bib67] Nebenführ A., Frohlick J.A., Staehelin L.A. (2000). Redistribution of Golgi stacks and other organelles during mitosis and cytokinesis in plant cells. Plant Physiol..

[bib68] Nishihama R., Ishikawa M., Araki S., Soyano T., Asada T., Machida Y. (2001). The NPK1 mitogen-activated protein kinase kinase kinase is a regulator of cell-plate formation in plant cytokinesis. Genes Dev..

[bib69] Nishihama R., Machida Y. (2001). Expansion of the phragmoplast during plant cytokinesis: a MAPK pathway may MAP it out. Curr. Opin. Plant Biol..

[bib70] Nunes V., Dantas M., Castro D., Vitiello E., Wang I., Carpi N., Balland M., Piel M., Aguiar P., Maiato H. (2020). Centrosome–nuclear axis repositioning drives the assembly of a bipolar spindle scaffold to ensure mitotic fidelity. Mole. Biol. Cell.

[bib71] Otegui M.S., Staehelin L.A. (2004). Electron tomographic analysis of post-meiotic cytokinesis during pollen development in *Arabidopsis thaliana*. Planta.

[bib72] Pan R., Lee Y.R.J., Liu B. (2004). Localization of two homologous *Arabidopsis* kinesin-related proteins in the phragmoplast. Planta.

[bib73] Pardo M., Nurse P. (2003). Equatorial retention of the contractile actin ring by microtubules during cytokinesis. Science.

[bib74] Petrovská B., Cenklová V., Pochylová Ž., Kourová H., Doskočilová A., Plíhal O., Binarová L., Binarová P. (2012). Plant Aurora kinases play a role in maintenance of primary meristems and control of endoreduplication. New Phytol..

[bib75] Petrovská B., Jeřábková H., Kohoutová L., Cenklová V., Pochylová Ž., Gelová Z., Kočárová G., Váchová L., Kurejová M., Tomaštíková E. (2013). Overexpressed TPX2 causes ectopic formation of microtubular arrays in the nuclei of acentrosomal plant cells. J. Exp. Botany.

[bib76] Pietra S., Gustavsson A., Kiefer C., Kalmbach L., Hörstedt P., Ikeda Y., Stepanova A.N., Alonso J.M., Grebe M. (2013). *Arabidopsis* SABRE and CLASP interact to stabilize cell division plane orientation and planar polarity. Nat. Commun..

[bib77] Porceddu A., Stals H., Reichheld J.P., Segers G., De Veylder L., de Pinho Barrôco R., Casteels P., Van Montagu M., Inzé D., Mironov V. (2001). A plant-specific cyclin-dependent kinase is involved in the control of G2/M progression in plants. J. Biol. Chem..

[bib78] Qi X., Kaneda M., Chen J., Geitmann A., Zheng H. (2011). A specific role for *Arabidopsis* TRAPPII in post-Golgi trafficking that is crucial for cytokinesis and cell polarity. Plant J..

[bib79] Rasmussen C.G., Humphries J.A., Smith L.G. (2011). Determination of symmetric and asymmetric division planes in plant cells. Annu. Rev. Plant Biol..

[bib80] Sano T., Higaki T., Oda Y., Hayashi T., Hasezawa S. (2005). Appearance of actin microfilament ‘twin peaks’ in mitosis and their function in cell plate formation, as visualized in tobacco BY-2 cells expressing GFP–fimbrin. Plant J.

[bib81] Sasabe M., Boudolf V., De Veylder L., Inzé D., Genschik P., Machida Y. (2011). Phosphorylation of a mitotic kinesin-like protein and a MAPKKK by cyclin-dependent kinases (CDKs) is involved in the transition to cytokinesis in plants. Proc. Nat. Acad. Sci..

[bib82] Sasabe M., Kosetsu K., Hidaka M., Murase A., Machida Y. (2011). *Arabidopsis thaliana* MAP65-1 and MAP65-2 function redundantly with MAP65-3/PLEIADE in cytokinesis downstream of MPK4. Plant Signal Behav..

[bib83] Sasabe M., Soyano T., Takahashi Y., Sonobe S., Igarashi H., Itoh T.J., Hidaka M., Machida Y. (2006). Phosphorylation of NtMAP65-1 by a MAP kinase down-regulates its activity of microtubule bundling and stimulates progression of cytokinesis of tobacco cells. Genes Dev..

[bib84] Schaefer E., Belcram K., Uyttewaal M., Duroc Y., Goussot M., Legland D., Laruelle E., de Tauzia-Moreau M.L., Pastuglia M., Bouchez D. (2017). The preprophase band of microtubules controls the robustness of division orientation in plants. Science.

[bib85] Segui-Simarro J.M., Austin J.R., White E.A., Staehelin L.A. (2004). Electron tomographic analysis of somatic cell plate formation in meristematic cells of *Arabidopsis* preserved by high-pressure freezing[W]. Plant Cell.

[bib86] Seltzer V., Janski N., Canaday J., Herzog E., Erhardt M., Evrard J.L., Schmit A.C. (2007). *Arabidopsis* GCP2 and GCP3 are part of a soluble $\gamma$-tubulin complex and have nuclear envelope targeting domains. Plant J..

[bib87] Shrestha S., Wilmeth L.J., Eyer J., Shuster C.B. (2012). PRC1 controls spindle polarization and recruitment of cytokinetic factors during monopolar cytokinesis. Mol. Biol. Cell.

[bib88] Sinclair R., Hsu G., Davis D., Chang M., Rosquete M., Iwasa J.H., Drakakaki G. (2022). Plant cytokinesis and the construction of new cell wall. FEBS Lett..

[bib89] Smertenko A. (2018). Phragmoplast expansion: the four-stroke engine that powers plant cytokinesis. Curr. Opin. Plant Biol..

[bib90] Smertenko A.P., Chang H.Y., Sonobe S., Fenyk S.I., Weingartner M., Bögre L., Hussey P.J. (2006). Control of the AtMAP65-1 interaction with microtubules through the cell cycle. J. Cell Sci..

[bib91] Smertenko A.P., Chang H.Y., Wagner V., Kaloriti D., Fenyk S., Sonobe S., Lloyd C., Hauser M.T., Hussey P.J. (2004). The *Arabidopsis* microtubule-associated protein AtMAP65-1: molecular analysis of its microtubule bundling activity. Plant Cell.

[bib92] Smertenko A.P., Hewitt S.L., Jacques C.N., Kacprzyk R., Liu Y., Marcec M.J., Moyo L., Ogden A., Oung H.M., Schmidt S. (2018). Phragmoplast microtubule dynamics—a game of zones. J. Cell Sci..

[bib93] Smertenko A.P., Piette B., Hussey P.J. (2011). The origin of phragmoplast asymmetry. Curr. Biol..

[bib94] Smertenko A., Saleh N., Igarashi H., Mori H., Hauser-Hahn I., Jiang C.-J., Sonobe S., Lloyd C.W., Hussey P.J. (2000). A new class of microtubule-associated proteins in plants. Nat. Cell Biol..

[bib95] Smith L.G., Gerttula S.M., Han S., Levy J. (2001). Tangled1: a microtubule binding protein required for the spatial control of cytokinesis in maize. J. Cell Biol..

[bib96] Smith L.G., Hake S., Sylvester A.W. (1996). The *tangled-1* mutation alters cell division orientations throughout maize leaf development without altering leaf shape. Development.

[bib97] Soyano T., Nishihama R., Morikiyo K., Ishikawa M., Machida Y. (2003). NQK1/NtMEK1 is a MAPKK that acts in the NPK1 MAPKKK-mediated MAPK cascade and is required for plant cytokinesis. Genes Dev..

[bib98] Spinner L., Gadeyne A., Belcram K., Goussot M., Moison M., Duroc Y., Eeckhout D., De Winne N., Schaefer E., Van De Slijke E. (2013). A protein phosphatase 2A complex spatially controls plant cell division. Nat. Commun..

[bib99] Steiner A., Rybak K., Altmann M., McFarlane H.E., Klaeger S., Nguyen N., Facher E., Ivakov A., Wanner G., Kuster B. (2016). Cell cycle-regulated PLEIADE/AtMAP65-3 links membrane and microtubule dynamics during plant cytokinesis. Plant J..

[bib100] Swan K.A., Severson A.F., Carter J.C., Martin P.R., Schnabel H., Schnabel R., Bowerman B. (1998). cyk-1: a *C. elegans* FH gene required for a late step in embryonic cytokinesis. J. Cell Sci..

[bib101] Takahashi Y., Soyano T., Kosetsu K., Sasabe M., Machida Y. (2010). HINKEL kinesin, ANP MAPKKKs and MKK6/ANQ MAPKK, which phosphorylates and activates MPK4 MAPK, constitute a pathway that is required for cytokinesis in *Arabidopsis thaliana*. Plant Cell Physiol..

[bib102] Tanaka H., Ishikawa M., Kitamura S., Takahashi Y., Soyano T., Machida C., Machida Y. (2004). The AtNACK1/HINKEL and STUD/TETRASPORE/AtNACK2 genes, which encode functionally redundant kinesins, are essential for cytokinesis in *Arabidopsis*. Genes Cells.

[bib103] Tena G., Asai T., Chiu W.L., Sheen J. (2001). Plant mitogen-activated protein kinase signaling cascades. Curr. Opin. Plant Biol..

[bib104] Thawani A., Kadzik R.S., Petry S. (2018). XMAP215 is a microtubule nucleation factor that functions synergistically with the $\gamma$-tubulin ring complex. Nat. Cell Biol..

[bib105] Thompson H.M., Skop A.R., Euteneuer U., Meyer B.J., McNiven M.A. (2002). The large GTPase dynamin associates with the spindle midzone and is required for cytokinesis. Curr. Biol..

[bib106] Twell D., Park S.K., Hawkins T.J., Schubert D., Schmidt R., Smertenko A., Hussey P.J. (2002). MOR1/GEM1 has an essential role in the plant-specific cytokinetic phragmoplast. Nat. Cell Biol..

[bib107] Van Damme D., Geelen D. (2008). Demarcation of the cortical division zone in dividing plant cells. Cell Biol. Int..

[bib108] Van Damme D., De Rybel B., Gudesblat G., Demidov D., Grunewald W., De Smet I., Houben A., Beeckman T., Russinova E. (2011). *Arabidopsis* $\alpha$ Aurora kinases function in formative cell division plane orientation. Plant Cell.

[bib109] Van Damme D., Van Poucke K., Boutant E., Ritzenthaler C., Inzé D., Geelen D. (2004). In vivo dynamics and differential microtubule-binding activities of MAP65 proteins. Plant Physiol..

[bib110] Vanstraelen M., Torres Acosta J.A., De Veylder L., Inzé D., Geelen D. (2004). A plant-specific subclass of C-terminal kinesins contains a conserved A-type cyclin-dependent kinase site implicated in folding and dimerization. Plant Physiol..

[bib111] Vanstraelen M., Van Damme D., De Rycke R., Mylle E., Inzé D., Geelen D. (2006). Cell cycle-dependent targeting of a kinesin at the plasma membrane demarcates the division site in plant cells. Curr. Biol..

[bib112] Walker K.L., Müller S., Moss D., Ehrhardt D.W., Smith L.G. (2007). *Arabidopsis* TANGLED identifies the division plane throughout mitosis and cytokinesis. Curr. Biol..

[bib113] Weingartner M., Criqui M.C., Meészaéros T., Binarova P., Schmit A.C., Helfer A., Derevier A., Erhardt M., Bögre L., Genschik P. (2004). Expression of a nondegradable cyclin B1 affects plant development and leads to endomitosis by inhibiting the formation of a phragmoplast. Plant Cell.

[bib114] Whittington A.T., Vugrek O., Wei K.J., Hasenbein N.G., Sugimoto K., Rashbrooke M.C., Wasteneys G.O. (2001). MOR1 is essential for organizing cortical microtubules in plants. Nature.

[bib115] Wu J., Tan X., Wu C., Cao K., Li Y., Bao Y. (2013). Regulation of cytokinesis by exocyst subunit SEC6 and KEULE in *Arabidopsis thaliana*. Mole. Plant.

[bib116] Xu J., Zhang S. (2015). Mitogen-activated protein kinase cascades in signaling plant growth and development. Trends Plant Sci..

[bib117] Zhang H., Deng X., Sun B., Lee Van S., Kang Z., Lin H., Lee Y.J., Liu B. (2018). Role of the BUB3 protein in phragmoplast microtubule reorganization during cytokinesis. Nat. Plants.

[bib118] Zhang L., Zhang H., Liu P., Hao H., Jin J.B., Lin J. (2011). *Arabidopsis* R-SNARE proteins VAMP721 and VAMP722 are required for cell plate formation. PLoS One.

[bib119] Zhang M., Zhang S. (2022). Mitogen-activated protein kinase cascades in plant signaling. J Integr Plant Biol.

